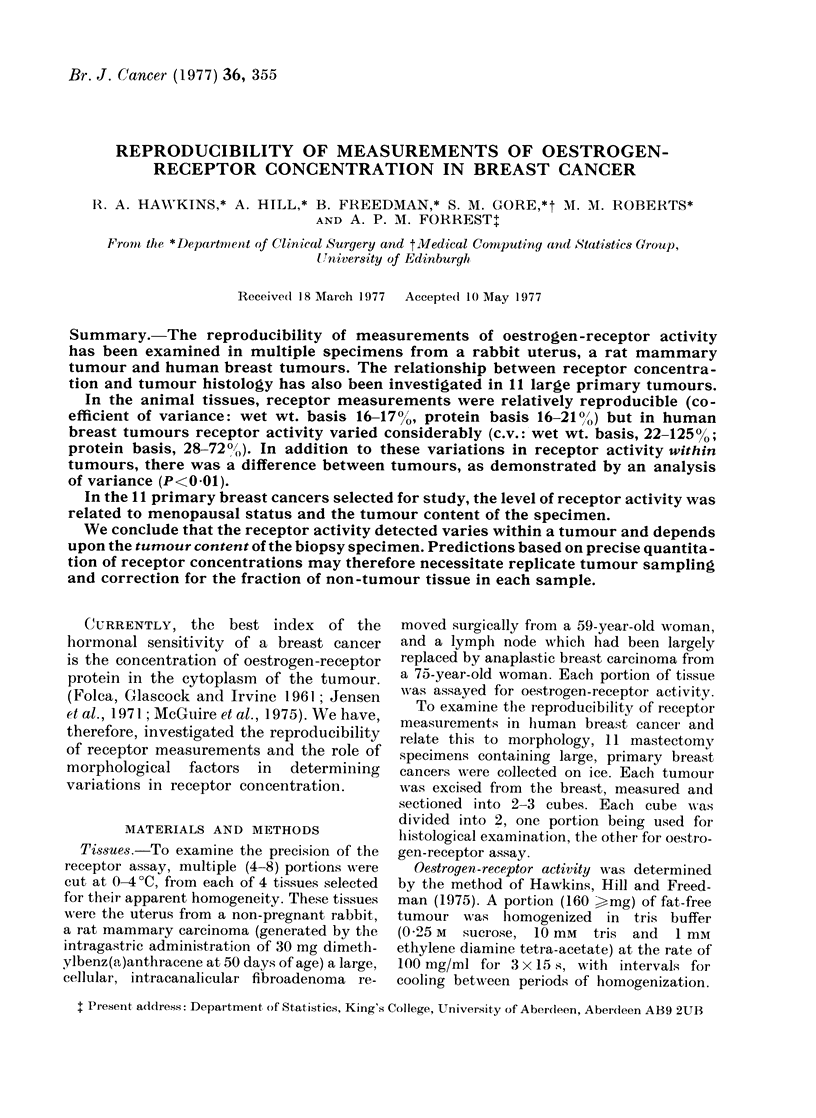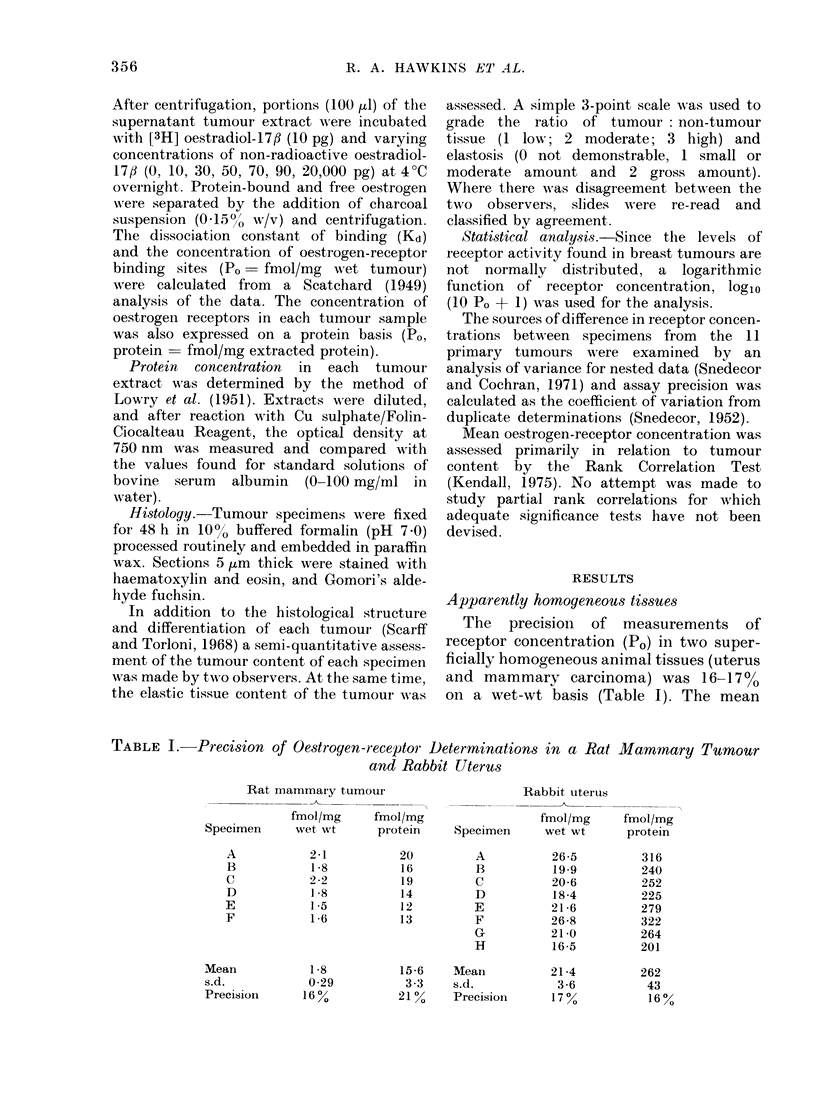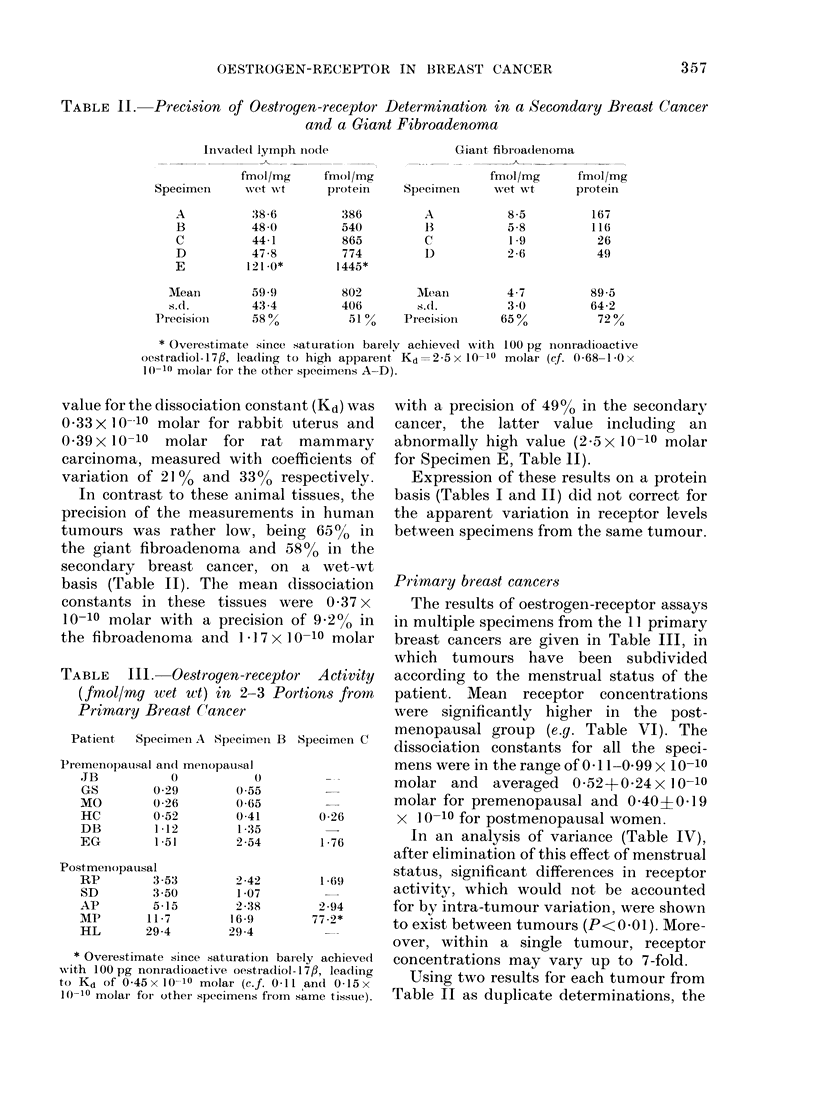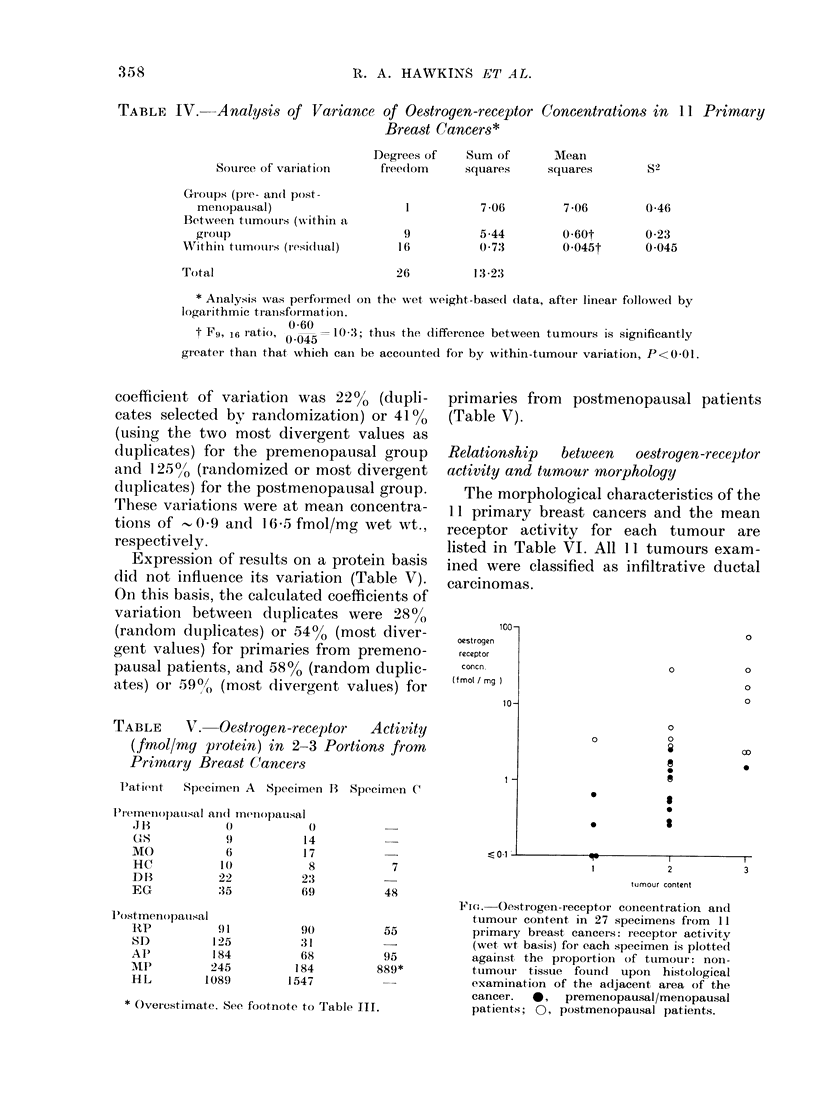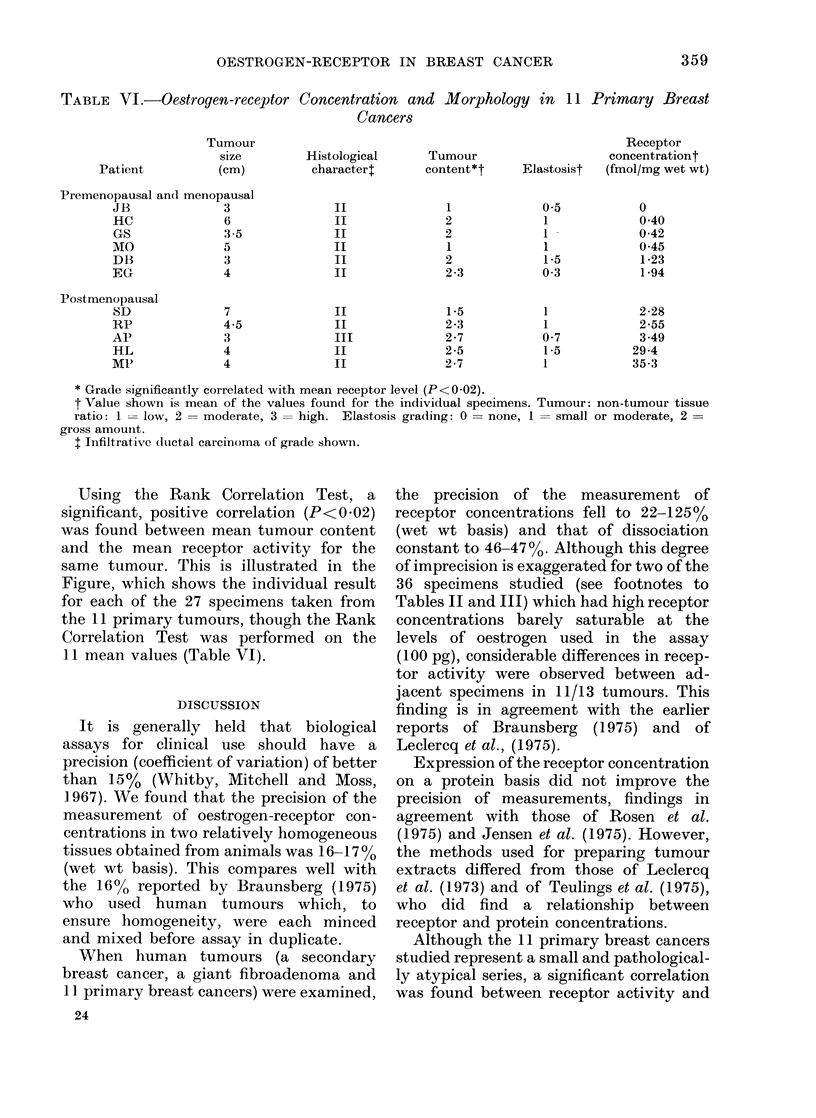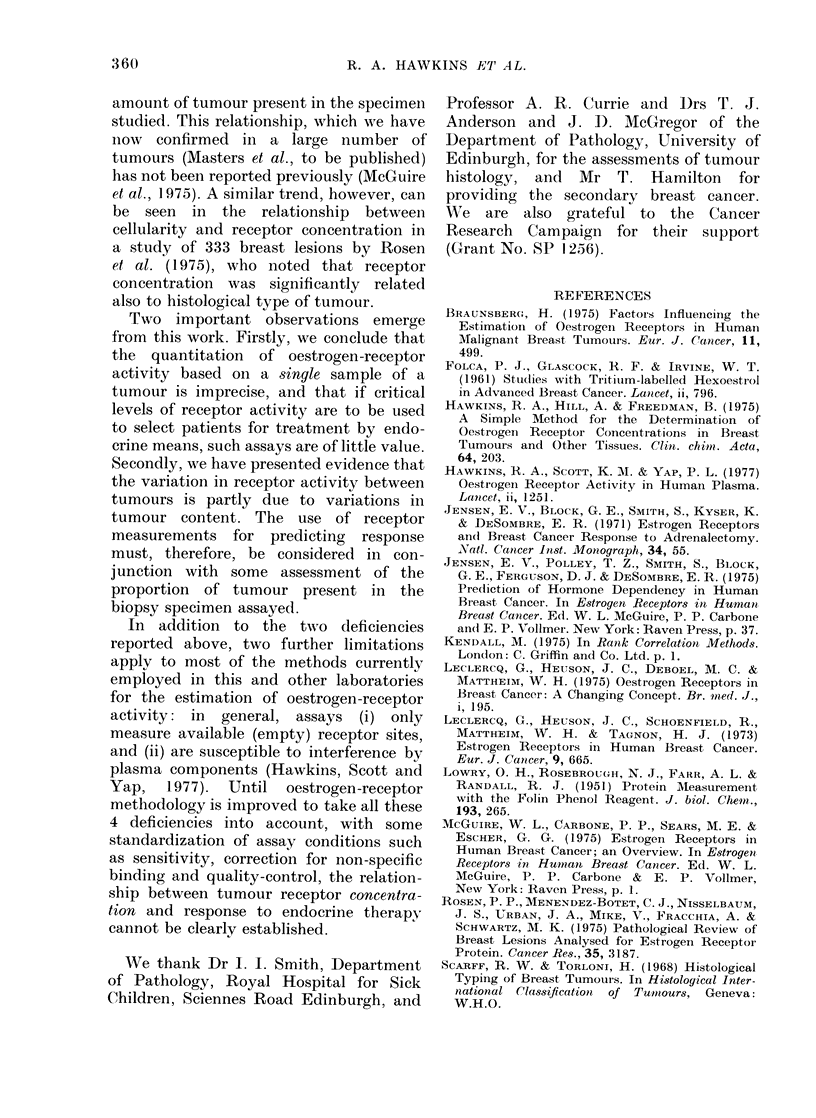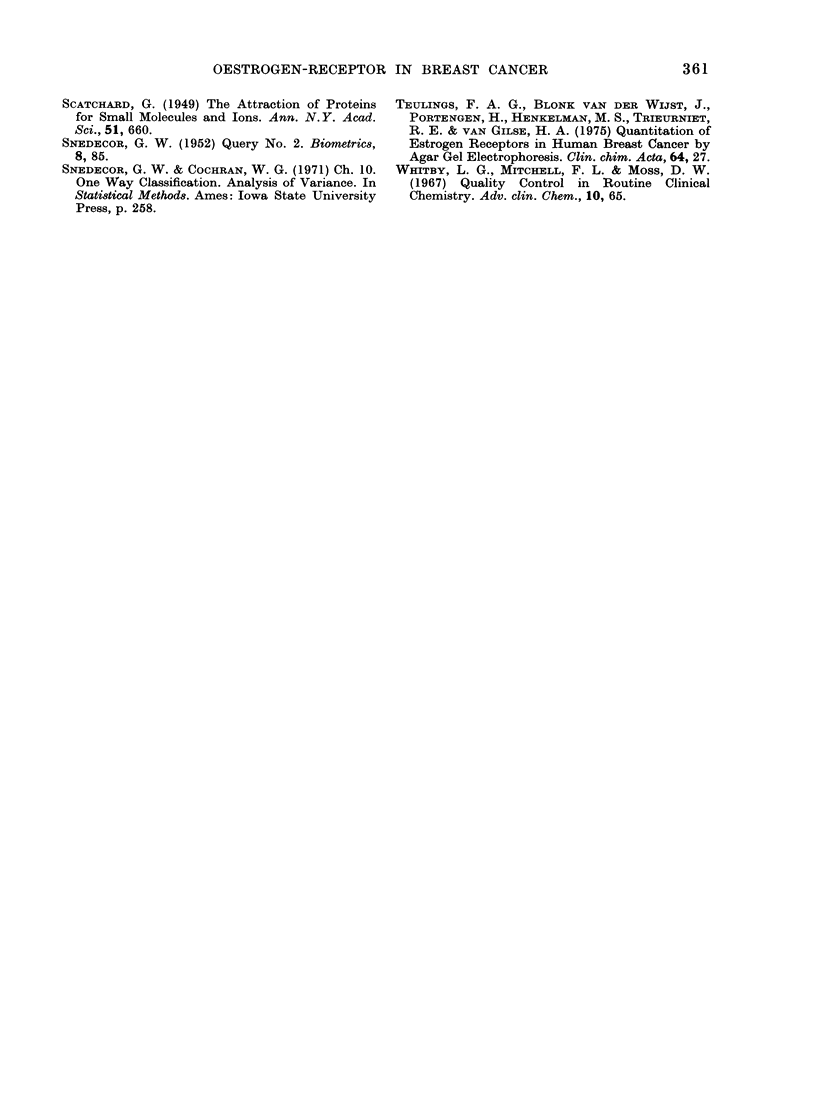# Reproducibility of measurements of oestrogen-receptor concentration in breast cancer.

**DOI:** 10.1038/bjc.1977.200

**Published:** 1977-09

**Authors:** R. A. Hawkins, A. Hill, B. Freedman, S. M. Gore, M. M. Roberts, A. P. Forrest

## Abstract

The reproducibility of measurements of oestrogen-receptor activity has been examined in multiple specimens from a rabbit uterus, a rat mammary tumour and human breast tumours. The relationship between receptor concentration and tumour histology has also been investigated in 11 large primary tumours. In the animal tissues, receptor measurements were relatively reproducible (coefficient of variance: wet wt. basis 16-17%, protein basis 16-21%) but in human breast tumours receptor activity varied considerably (c.v.: wet wt. basis, 22-125%; protein basis, 28-72%). In addition to these variations in receptor activity within tumours, there was a difference between tumours, as demonstrated by an analysis of variance (P less than 0.01). In the 11 primary breast cancers selected for study, the level of receptor activity was related to menopausal status and the tumour content of the specimen. We conclude that the receptor activity detected varies within a tumour and depends upon the tumour content of the biopsy specimen. Predictions based on precise quantitation of receptor concentrations may therefore necessitate replicate tumour sampling and correction for the fraction of non-tumour tissue in each sample.


*Br. J. Cancer* (1977) **36**, 355

### REPRODUCIBILITY OF MEASUREMENTS OF OESTROGEN-RECEPTOR CONCENTRATION IN BREAST CANCER

R. A. HAWKINS,* A. HILL,* B. FREEDMAN,* S. M. GORE,*† M. M. ROBERTS* AND A. P. M. FORREST‡

*From the *Department of Clinical Surgery and †Medical Computing and Statistics Group, University of Edinburgh*

Received 18 March 1977 Accepted 10 May 1977

**Summary.—The reproducibility of measurements of oestrogen-receptor activity has been examined in multiple specimens from a rabbit uterus, a rat mammary tumour and human breast tumours. The relationship between receptor concentration and tumour histology has also been investigated in 11 large primary tumours.**

**In the animal tissues, receptor measurements were relatively reproducible (coefficient of variance: wet wt. basis 16–17%, protein basis 16–21%) but in human breast tumours receptor activity varied considerably (c.v.: wet wt. basis, 22–125%; protein basis, 28–72%). In addition to these variations in receptor activity *within* tumours, there was a difference between tumours, as demonstrated by an analysis of variance ($P<0{\cdot}01$).**

**In the 11 primary breast cancers selected for study, the level of receptor activity was related to menopausal status and the tumour content of the specimen.**

**We conclude that the receptor activity detected varies within a tumour and depends upon the *tumour content* of the biopsy specimen. Predictions based on precise quantitation of receptor concentrations may therefore necessitate replicate tumour sampling and correction for the fraction of non-tumour tissue in each sample.**

CURRENTLY, the best index of the hormonal sensitivity of a breast cancer is the concentration of oestrogen-receptor protein in the cytoplasm of the tumour. (Folca, Glascock and Irvine 1961; Jensen *et al.*, 1971; McGuire *et al.*, 1975). We have, therefore, investigated the reproducibility of receptor measurements and the role of morphological factors in determining variations in receptor concentration.

### MATERIALS AND METHODS

*Tissues.*—To examine the precision of the receptor assay, multiple (4–8) portions were cut at 0–4 °C, from each of 4 tissues selected for their apparent homogeneity. These tissues were the uterus from a non-pregnant rabbit, a rat mammary carcinoma (generated by the intragastric administration of 30 mg dimethylbenz(a)anthracene at 50 days of age) a large, cellular, intracanalicular fibroadenoma removed surgically from a 59-year-old woman, and a lymph node which had been largely replaced by anaplastic breast carcinoma from a 75-year-old woman. Each portion of tissue was assayed for oestrogen-receptor activity.

To examine the reproducibility of receptor measurements in human breast cancer and relate this to morphology, 11 mastectomy specimens containing large, primary breast cancers were collected on ice. Each tumour was excised from the breast, measured and sectioned into 2–3 cubes. Each cube was divided into 2, one portion being used for histological examination, the other for oestrogen-receptor assay.

*Oestrogen-receptor activity* was determined by the method of Hawkins, Hill and Freedman (1975). A portion (160 ⩾mg) of fat-free tumour was homogenized in tris buffer (0·25 M sucrose, 10 mM tris and 1 mM ethylene diamine tetra-acetate) at the rate of 100 mg/ml for 3 × 15 s, with intervals for cooling between periods of homogenization.

‡ Present address: Department of Statistics, King's College, University of Aberdeen, Aberdeen AB9 2UB

After centrifugation, portions (100 $\mu$l) of the supernatant tumour extract were incubated with [$^3$H] oestradiol-17$\beta$ (10 pg) and varying concentrations of non-radioactive oestradiol-17$\beta$ (0, 10, 30, 50, 70, 90, 20,000 pg) at 4°C overnight. Protein-bound and free oestrogen were separated by the addition of charcoal suspension (0·15% w/v) and centrifugation. The dissociation constant of binding ($K_d$) and the concentration of oestrogen-receptor binding sites ($P_o$ = fmol/mg wet tumour) were calculated from a Scatchard (1949) analysis of the data. The concentration of oestrogen receptors in each tumour sample was also expressed on a protein basis ($P_o$, protein = fmol/mg extracted protein).

*Protein concentration* in each tumour extract was determined by the method of Lowry *et al.* (1951). Extracts were diluted, and after reaction with Cu sulphate/Folin-Ciocalteau Reagent, the optical density at 750 nm was measured and compared with the values found for standard solutions of bovine serum albumin (0–100 mg/ml in water).

*Histology.*—Tumour specimens were fixed for 48 h in 10% buffered formalin (pH 7·0) processed routinely and embedded in paraffin wax. Sections 5 $\mu$m thick were stained with haematoxylin and eosin, and Gomori's aldehyde fuchsin.

In addition to the histological structure and differentiation of each tumour (Scarff and Torloni, 1968) a semi-quantitative assessment of the tumour content of each specimen was made by two observers. At the same time, the elastic tissue content of the tumour was assessed. A simple 3-point scale was used to grade the ratio of tumour : non-tumour tissue (1 low; 2 moderate; 3 high) and elastosis (0 not demonstrable, 1 small or moderate amount and 2 gross amount). Where there was disagreement between the two observers, slides were re-read and classified by agreement.

*Statistical analysis.*—Since the levels of receptor activity found in breast tumours are not normally distributed, a logarithmic function of receptor concentration, $\log_{10}(10\ P_o + 1)$ was used for the analysis.

The sources of difference in receptor concentrations between specimens from the 11 primary tumours were examined by an analysis of variance for nested data (Snedecor and Cochran, 1971) and assay precision was calculated as the coefficient of variation from duplicate determinations (Snedecor, 1952).

Mean oestrogen-receptor concentration was assessed primarily in relation to tumour content by the Rank Correlation Test (Kendall, 1975). No attempt was made to study partial rank correlations for which adequate significance tests have not been devised.

### RESULTS

### *Apparently homogeneous tissues*

The precision of measurements of receptor concentration ($P_0$) in two superficially homogeneous animal tissues (uterus and mammary carcinoma) was 16–17% on a wet-wt basis (Table I). The mean

TABLE I.—*Precision of Oestrogen-receptor Determinations in a Rat Mammary Tumour and Rabbit Uterus*

| Rat mammary tumour | | | Rabbit uterus | | |
|---|---|---|---|---|---|
| Specimen | fmol/mg wet wt | fmol/mg protein | Specimen | fmol/mg wet wt | fmol/mg protein |
| A | 2·1 | 20 | A | 26·5 | 316 |
| B | 1·8 | 16 | B | 19·9 | 240 |
| C | 2·2 | 19 | C | 20·6 | 252 |
| D | 1·8 | 14 | D | 18·4 | 225 |
| E | 1·5 | 12 | E | 21·6 | 279 |
| F | 1·6 | 13 | F | 26·8 | 322 |
| | | | G | 21·0 | 264 |
| | | | H | 16·5 | 201 |
| Mean | 1·8 | 15·6 | Mean | 21·4 | 262 |
| s.d. | 0·29 | 3·3 | s.d. | 3·6 | 43 |
| Precision | 16% | 21% | Precision | 17% | 16% |

TABLE II.—*Precision of Oestrogen-receptor Determination in a Secondary Breast Cancer and a Giant Fibroadenoma*

| Invaded lymph node | | | Giant fibroadenoma | | |
|---|---|---|---|---|---|
| Specimen | fmol/mg wet wt | fmol/mg protein | Specimen | fmol/mg wet wt | fmol/mg protein |
| A | 38·6 | 386 | A | 8·5 | 167 |
| B | 48·0 | 540 | B | 5·8 | 116 |
| C | 44·1 | 865 | C | 1·9 | 26 |
| D | 47·8 | 774 | D | 2·6 | 49 |
| E | 121·0* | 1445* | | | |
| Mean | 59·9 | 802 | Mean | 4·7 | 89·5 |
| s.d. | 43·4 | 406 | s.d. | 3·0 | 64·2 |
| Precision | 58% | 51% | Precision | 65% | 72% |

* Overestimate since saturation barely achieved with 100 pg nonradioactive oestradiol-17$\beta$, leading to high apparent $K_d = 2{\cdot}5 \times 10^{-10}$ molar (*cf.* $0{\cdot}68$–$1{\cdot}0 \times 10^{-10}$ molar for the other specimens A–D).

value for the dissociation constant ($K_d$) was $0{\cdot}33 \times 10^{-10}$ molar for rabbit uterus and $0{\cdot}39 \times 10^{-10}$ molar for rat mammary carcinoma, measured with coefficients of variation of 21% and 33% respectively.

In contrast to these animal tissues, the precision of the measurements in human tumours was rather low, being 65% in the giant fibroadenoma and 58% in the secondary breast cancer, on a wet-wt basis (Table II). The mean dissociation constants in these tissues were $0{\cdot}37 \times 10^{-10}$ molar with a precision of 9·2% in the fibroadenoma and $1{\cdot}17 \times 10^{-10}$ molar with a precision of 49% in the secondary cancer, the latter value including an abnormally high value ($2{\cdot}5 \times 10^{-10}$ molar for Specimen E, Table II).

Expression of these results on a protein basis (Tables I and II) did not correct for the apparent variation in receptor levels between specimens from the same tumour.

TABLE III.—*Oestrogen-receptor Activity (fmol/mg wet wt) in 2–3 Portions from Primary Breast Cancer*

| Patient | Specimen A | Specimen B | Specimen C |
|---|---|---|---|
| Premenopausal and menopausal | | | |
| JB | 0 | 0 | — |
| GS | 0·29 | 0·55 | — |
| MO | 0·26 | 0·65 | — |
| HC | 0·52 | 0·41 | 0·26 |
| DB | 1·12 | 1·35 | — |
| EG | 1·51 | 2·54 | 1·76 |
| Postmenopausal | | | |
| RP | 3·53 | 2·42 | 1·69 |
| SD | 3·50 | 1·07 | — |
| AP | 5·15 | 2·38 | 2·94 |
| MP | 11·7 | 16·9 | 77·2* |
| HL | 29·4 | 29·4 | — |

* Overestimate since saturation barely achieved with 100 pg nonradioactive oestradiol-17$\beta$, leading to $K_d$ of $0{\cdot}45 \times 10^{-10}$ molar (*c.f.* 0·11 and $0{\cdot}15 \times 10^{-10}$ molar for other specimens from same tissue).

*Primary breast cancers*

The results of oestrogen-receptor assays in multiple specimens from the 11 primary breast cancers are given in Table III, in which tumours have been subdivided according to the menstrual status of the patient. Mean receptor concentrations were significantly higher in the post-menopausal group (*e.g.* Table VI). The dissociation constants for all the specimens were in the range of $0{\cdot}11$–$0{\cdot}99 \times 10^{-10}$ molar and averaged $0{\cdot}52 + 0{\cdot}24 \times 10^{-10}$ molar for premenopausal and $0{\cdot}40 \pm 0{\cdot}19 \times 10^{-10}$ for postmenopausal women.

In an analysis of variance (Table IV), after elimination of this effect of menstrual status, significant differences in receptor activity, which would not be accounted for by intra-tumour variation, were shown to exist between tumours ($P < 0{\cdot}01$). Moreover, within a single tumour, receptor concentrations may vary up to 7-fold.

Using two results for each tumour from Table II as duplicate determinations, the

TABLE IV.—*Analysis of Variance of Oestrogen-receptor Concentrations in 11 Primary Breast Cancers**

| Source of variation | Degrees of freedom | Sum of squares | Mean squares | $S^2$ |
|---|---|---|---|---|
| Groups (pre- and post-menopausal) | 1 | 7·06 | 7·06 | 0·46 |
| Between tumours (within a group | 9 | 5·44 | 0·60† | 0·23 |
| Within tumours (residual) | 16 | 0·73 | 0·045† | 0·045 |
| Total | 26 | 13·23 | | |

* Analysis was performed on the wet weight-based data, after linear followed by logarithmic transformation.

† $F_{9,16}$ ratio, $\frac{0 \cdot 60}{0 \cdot 045} = 10 \cdot 3$; thus the difference between tumours is significantly greater than that which can be accounted for by within-tumour variation, $P < 0 \cdot 01$.

coefficient of variation was 22% (duplicates selected by randomization) or 41% (using the two most divergent values as duplicates) for the premenopausal group and 125% (randomized or most divergent duplicates) for the postmenopausal group. These variations were at mean concentrations of ~0·9 and 16·5 fmol/mg wet wt., respectively.

Expression of results on a protein basis did not influence its variation (Table V). On this basis, the calculated coefficients of variation between duplicates were 28% (random duplicates) or 54% (most divergent values) for primaries from premenopausal patients, and 58% (random duplicates) or 59% (most divergent values) for primaries from postmenopausal patients (Table V).

TABLE V.—*Oestrogen-receptor Activity (fmol/mg protein) in 2–3 Portions from Primary Breast Cancers*

| Patient | Specimen A | Specimen B | Specimen C |
|---|---|---|---|
| Premenopausal and menopausal | | | |
| JB | 0 | 0 | — |
| GS | 9 | 14 | — |
| MO | 6 | 17 | — |
| HC | 10 | 8 | 7 |
| DB | 22 | 23 | — |
| EG | 35 | 69 | 48 |
| Postmenopausal | | | |
| RP | 91 | 90 | 55 |
| SD | 125 | 31 | — |
| AP | 184 | 68 | 95 |
| MP | 245 | 184 | 889* |
| HL | 1089 | 1547 | — |

* Overestimate. See footnote to Table III.

*Relationship between oestrogen-receptor activity and tumour morphology*

The morphological characteristics of the 11 primary breast cancers and the mean receptor activity for each tumour are listed in Table VI. All 11 tumours examined were classified as infiltrative ductal carcinomas.

FIG.—Oestrogen-receptor concentration and tumour content in 27 specimens from 11 primary breast cancers: receptor activity (wet wt basis) for each specimen is plotted against the proportion of tumour: non-tumour tissue found upon histological examination of the adjacent area of the cancer. ●, premenopausal/menopausal patients; ○, postmenopausal patients.

TABLE VI.—*Oestrogen-receptor Concentration and Morphology in* 11 *Primary Breast Cancers*

| Patient | Tumour size (cm) | Histological character‡ | Tumour content*† | Elastosis† | Receptor concentration† (fmol/mg wet wt) |
|---|---|---|---|---|---|
| Premenopausal and menopausal | | | | | |
| JB | 3 | II | 1 | 0·5 | 0 |
| HC | 6 | II | 2 | 1 | 0·40 |
| GS | 3·5 | II | 2 | 1 | 0·42 |
| MO | 5 | II | 1 | 1 | 0·45 |
| DB | 3 | II | 2 | 1·5 | 1·23 |
| EG | 4 | II | 2·3 | 0·3 | 1·94 |
| Postmenopausal | | | | | |
| SD | 7 | II | 1·5 | 1 | 2·28 |
| RP | 4·5 | II | 2·3 | 1 | 2·55 |
| AP | 3 | III | 2·7 | 0·7 | 3·49 |
| HL | 4 | II | 2·5 | 1·5 | 29·4 |
| MP | 4 | II | 2·7 | 1 | 35·3 |

* Grade significantly correlated with mean receptor level ($P<0{\cdot}02$).
† Value shown is mean of the values found for the individual specimens. Tumour: non-tumour tissue ratio: 1 = low, 2 = moderate, 3 = high. Elastosis grading: 0 = none, 1 = small or moderate, 2 = gross amount.
‡ Infiltrative ductal carcinoma of grade shown.

Using the Rank Correlation Test, a significant, positive correlation ($P<0{\cdot}02$) was found between mean tumour content and the mean receptor activity for the same tumour. This is illustrated in the Figure, which shows the individual result for each of the 27 specimens taken from the 11 primary tumours, though the Rank Correlation Test was performed on the 11 mean values (Table VI).

### DISCUSSION

It is generally held that biological assays for clinical use should have a precision (coefficient of variation) of better than 15% (Whitby, Mitchell and Moss, 1967). We found that the precision of the measurement of oestrogen-receptor concentrations in two relatively homogeneous tissues obtained from animals was 16–17% (wet wt basis). This compares well with the 16% reported by Braunsberg (1975) who used human tumours which, to ensure homogeneity, were each minced and mixed before assay in duplicate.

When human tumours (a secondary breast cancer, a giant fibroadenoma and 11 primary breast cancers) were examined, the precision of the measurement of receptor concentrations fell to 22–125% (wet wt basis) and that of dissociation constant to 46–47%. Although this degree of imprecision is exaggerated for two of the 36 specimens studied (see footnotes to Tables II and III) which had high receptor concentrations barely saturable at the levels of oestrogen used in the assay (100 pg), considerable differences in receptor activity were observed between adjacent specimens in 11/13 tumours. This finding is in agreement with the earlier reports of Braunsberg (1975) and of Leclercq *et al.*, (1975).

Expression of the receptor concentration on a protein basis did not improve the precision of measurements, findings in agreement with those of Rosen *et al.* (1975) and Jensen *et al.* (1975). However, the methods used for preparing tumour extracts differed from those of Leclercq *et al.* (1973) and of Teulings *et al.* (1975), who did find a relationship between receptor and protein concentrations.

Although the 11 primary breast cancers studied represent a small and pathologically atypical series, a significant correlation was found between receptor activity and amount of tumour present in the specimen studied. This relationship, which we have now confirmed in a large number of tumours (Masters *et al.*, to be published) has not been reported previously (McGuire *et al.*, 1975). A similar trend, however, can be seen in the relationship between cellularity and receptor concentration in a study of 333 breast lesions by Rosen *et al.* (1975), who noted that receptor concentration was significantly related also to histological type of tumour.

Two important observations emerge from this work. Firstly, we conclude that the quantitation of oestrogen-receptor activity based on a *single* sample of a tumour is imprecise, and that if critical levels of receptor activity are to be used to select patients for treatment by endocrine means, such assays are of little value. Secondly, we have presented evidence that the variation in receptor activity between tumours is partly due to variations in tumour content. The use of receptor measurements for predicting response must, therefore, be considered in conjunction with some assessment of the proportion of tumour present in the biopsy specimen assayed.

In addition to the two deficiencies reported above, two further limitations apply to most of the methods currently employed in this and other laboratories for the estimation of oestrogen-receptor activity: in general, assays (i) only measure available (empty) receptor sites, and (ii) are susceptible to interference by plasma components (Hawkins, Scott and Yap, 1977). Until oestrogen-receptor methodology is improved to take all these 4 deficiencies into account, with some standardization of assay conditions such as sensitivity, correction for non-specific binding and quality-control, the relationship between tumour receptor *concentration* and response to endocrine therapy cannot be clearly established.

We thank Dr I. I. Smith, Department of Pathology, Royal Hospital for Sick Children, Sciennes Road Edinburgh, and Professor A. R. Currie and Drs T. J. Anderson and J. D. McGregor of the Department of Pathology, University of Edinburgh, for the assessments of tumour histology, and Mr T. Hamilton for providing the secondary breast cancer. We are also grateful to the Cancer Research Campaign for their support (Grant No. SP 1256).

### REFERENCES

BRAUNSBERG, H. (1975) Factors Influencing the Estimation of Oestrogen Receptors in Human Malignant Breast Tumours. *Eur. J. Cancer*, **11**, 499.

FOLCA, P. J., GLASCOCK, R. F. & IRVINE, W. T. (1961) Studies with Tritium-labelled Hexoestrol in Advanced Breast Cancer. *Lancet*, ii, 796.

HAWKINS, R. A., HILL, A. & FREEDMAN, B. (1975) A Simple Method for the Determination of Oestrogen Receptor Concentrations in Breast Tumours and Other Tissues. *Clin. chim. Acta*, **64**, 203.

HAWKINS, R. A., SCOTT, K. M. & YAP, P. L. (1977) Oestrogen Receptor Activity in Human Plasma. *Lancet*, ii, 1251.

JENSEN, E. V., BLOCK, G. E., SMITH, S., KYSER, K. & DESOMBRE, E. R. (1971) Estrogen Receptors and Breast Cancer Response to Adrenalectomy. *Natl. Cancer Inst. Monograph*, **34**, 55.

JENSEN, E. V., POLLEY, T. Z., SMITH, S., BLOCK, G. E., FERGUSON, D. J. & DESOMBRE, E. R. (1975) Prediction of Hormone Dependency in Human Breast Cancer. In *Estrogen Receptors in Human Breast Cancer*. Ed. W. L. McGuire, P. P. Carbone and E. P. Vollmer. New York: Raven Press, p. 37.

KENDALL, M. (1975) In *Rank Correlation Methods*. London: C. Griffin and Co. Ltd. p. 1.

LECLERCQ, G., HEUSON, J. C., DEBOEL, M. C. & MATTHEIM, W. H. (1975) Oestrogen Receptors in Breast Cancer: A Changing Concept. *Br. med. J.*, i, 195.

LECLERCQ, G., HEUSON, J. C., SCHOENFIELD, R., MATTHEIM, W. H. & TAGNON, H. J. (1973) Estrogen Receptors in Human Breast Cancer. *Eur. J. Cancer*, **9**, 665.

LOWRY, O. H., ROSEBROUGH, N. J., FARR, A. L. & RANDALL, R. J. (1951) Protein Measurement with the Folin Phenol Reagent. *J. biol. Chem.*, **193**, 265.

MCGUIRE, W. L., CARBONE, P. P., SEARS, M. E. & ESCHER, G. G. (1975) Estrogen Receptors in Human Breast Cancer; an Overview. In *Estrogen Receptors in Human Breast Cancer*. Ed. W. L. McGuire, P. P. Carbone & E. P. Vollmer, New York: Raven Press, p. 1.

ROSEN, P. P., MENENDEZ-BOTET, C. J., NISSELBAUM, J. S., URBAN, J. A., MIKE, V., FRACCHIA, A. & SCHWARTZ, M. K. (1975) Pathological Review of Breast Lesions Analysed for Estrogen Receptor Protein. *Cancer Res.*, **35**, 3187.

SCARFF, R. W. & TORLONI, H. (1968) Histological Typing of Breast Tumours. In *Histological International Classification of Tumours*, Geneva: W.H.O.

SCATCHARD, G. (1949) The Attraction of Proteins for Small Molecules and Ions. *Ann. N.Y. Acad. Sci.*, **51**, 660.

SNEDECOR, G. W. (1952) Query No. 2. *Biometrics*, **8**, 85.

SNEDECOR, G. W. & COCHRAN, W. G. (1971) Ch. 10. One Way Classification. Analysis of Variance. In *Statistical Methods*. Ames: Iowa State University Press, p. 258.

TEULINGS, F. A. G., BLONK VAN DER WIJST, J., PORTENGEN, H., HENKELMAN, M. S., TRIEURNIET, R. E. & VAN GILSE, H. A. (1975) Quantitation of Estrogen Receptors in Human Breast Cancer by Agar Gel Electrophoresis. *Clin. chim. Acta*, **64**, 27.

WHITBY, L. G., MITCHELL, F. L. & MOSS, D. W. (1967) Quality Control in Routine Clinical Chemistry. *Adv. clin. Chem.*, **10**, 65.